# Biological properties of valve materials using RGD and EC

**DOI:** 10.1515/med-2025-1195

**Published:** 2025-09-04

**Authors:** Chennian Xu, Yang Liu, Rui Qiao, Ping Jin, Hao Tang, Zhiyuan Tian, Bin Cui, Anguo Wen, Jian Yang

**Affiliations:** Key Laboratory of Gastrointestinal Pharmacology of Chinese Materia Medica of the State Administration of Traditional Chinese Medicine, Department of Pharmacology, School of Pharmacy, The Fourth Military Medical University, Xi’an, 710032, Shaanxi, China; Department of Cardiovascular Surgery, Xijing Hospital, The Fourth Military Medical University, 710032, Shaanxi, P. R. China; The 79th Group Military Hospital of the Chinese People’s Liberation Army, Liaoyang, 111000, Liaoning, China; Department of First Cadre Ward, General Hospital of the Northern Theater Command, Shenyang, 110016, Liaoning, China; Department of Gastroenterology, Hospital of the Northern Theater Command Air Force, Shenyang, 110092, Liaoning, China; Department of Cardiovascular Surgery, Xijing Hospital, The Fourth Military Medical University, No. 127 Changle West Rd, Xi’an, 710032, Shaanxi, P. R. China

**Keywords:** valvular heart disease, valve, biological properties, arginine-glycine-aspartate peptide, epoxy chloropropane

## Abstract

**Background:**

Rapid application of transcatheter valve replacement for valve diseases has promoted the development of biological valves. Different efforts have been made to improve the surface properties of valves. We developed a new method using arginine-glycine-aspartate (RGD) peptide and epoxy chloropropane (EC). Our goal was to evaluate and detect the surface biological characteristics of valve materials using RGD and EC treatments.

**Methods:**

The surfaces of the valve materials used in the GA-EC and RGD-EC groups were smooth and relatively dense, as observed using a scanning electron microscope.

**Results:**

More MSCs adhered to and grew on the samples in the GA-EC and RGD-EC groups than in the GA group. The apoptosis rate of MSCs was markedly decreased, whereas the expression of vimentin was elevated in the GA-EC and RGD-EC groups (*P* < 0.05). The adherent ability of MSCs in the RGD-EC and GA-EC groups was significantly higher than that in the GA group (*P* < 0.05).

**Conclusions:**

The new treatment method using RGD and EC improves the biological properties of the surface of the biological valve materials.

## 1 Introduction

The durability of biological valves is a key issue in the treatment of valvular heart disease. Valvular heart disease is the third most prevalent cardiovascular disease, second only to hypertension and coronary heart disease, and can cause arrhythmias, heart failure, vascular embolism, and other complications. This poses a serious threat to human health [1,2]. As people live longer and their eating habits change, the number of cases caused by degenerative valve diseases, such as calcific stenosis or insufficiency, is increasing [3,4,5,6,7]. Transcatheter valve replacement (TVR) has become an alternative treatment because of its lower risk and faster recovery in high-risk patients [8,9]. This will increase the frequency of use of biological valve materials to some extent. However, shortcomings of long-term durability and poor calcification exist in the clinical application of biological valves. As previously reported for porcine and other biological valves, cellular infiltration of the valve occurs in a time-dependent manner, and a longer implantation period may be needed for complete cellular infiltration to occur [10,11,12]. The anti-calcification processing and surface modification of cardiac biological valves have become the focus and direction in the field of artificial biological heart valve research [13,14,15,16,17]. However, the surface performance of the valves over time must be further improved.

Our study uses an RGD peptide on the GA surface and a bionic pulsating bioreactor to construct a tissue-engineered biological valve. It was designed to improve the properties of biological valve mechanics in terms of anti-calcification activity, biocompatibility, and long-term durability. Our study demonstrated a simple result on the new treatment method using RGD and EC improves the biological properties of the surface of the biological valve material.

## 2 Materials and methods

### 2.1 Materials

Four-week male healthy SD rats weighing 90–100 g were purchased from the Fourth Military Medical University (Xi’an, China) and kept under standard housing conditions. The animal experiments in this study were approved by the Animal Care Committee of the Fourth Military Medical University and conformed to the Principles of Laboratory Animal Care published by the US National Institutes of Health. The mice were housed in an environment with controlled humidity (55–60%) and temperature (22–25°C) and a controlled light cycle (12 h light and 12 h dark).

The following materials were used in the experiments: Dulbecco’s modified Eagle’s medium, a low-sugar culture medium (Thermo Fisher Scientific, Waltham, MA, USA); fetal bovine serum (Cytiva, HyClone Laboratories, Logan, Utah, USA), 0.25% trypsin (Cytiva, HyClone Laboratories), fluorescein isothiocyanate (FITC)-labeled goat anti-mouse CD44 antibody (Santa Cruz Biotechnology, Santa Cruz, CA, USA), and FITC-labeled goat anti-mouse CD45 antibody (Santa Cruz Biotechnology); FITC-labeled goat anti-mouse CD90 antibody (Abcam, Cambridge, UK), the terminal deoxynucleotidyl transferase deoxyuridine triphosphate nick end labeling (TUNEL) kit (F. Hoffmann-La Roche AG, Basel, Switzerland), glutaraldehyde (GA; Shanghai McLean Biochemical Technology Co., Ltd, China), epoxy chloropropane (EC, Shanghai McLean Biochemical Technology Co., Ltd, China), Dulbecco’s modified Eagle’s medium-low glucose (DMEM-LG, Thermofisher Scientific, the USA), and vimentin (Sigma-Aldrich, St. Louis, MO, USA). Arginine-glycine-aspartate was entrusted to Shanghai Shenggong Bioengineering Co., Ltd (China) for production.

All of the experiments were performed in the Department of Cardiovascular Surgery, Xijing Hospital, The Fourth Military Medical University.

### 2.2 GA treatment of biological valve materials

The bovine pericardium was used as the valve material and was exposed to warm ischemic conditions for 2 h. Fat and muscle tissues were removed using a 4D-Hanks balanced salt solution. The material was rinsed twice in phosphate-buffered saline (PBS). It was then soaked in a 0.3% GA solution at room temperature for 48 h. It was preserved in 0.5% GA solution and stored at 4°C for later use.

### 2.3 GA chloropropane treatment of the biological valve materials

The valve material that had been treated with GA was rinsed twice with PBS. It was then treated with an 8% EC solution for 48 h and rinsed twice with PBS. It was stored in 0.5% GA solution at 4°C until use.

### 2.4 Arginine-glycine-aspartate epichlorohydrin (RGD-EC) treatment of the valve materials

The valve material was treated with RGD peptide solution (1.5 g/L, Shanghai Shenggong Bioengineering Co., Ltd., China) for 24 h at room temperature at pH 7.4. The RGD-treated valve material was washed twice with PBS solution, treated with 8% EC solution for 48 h, and again washed twice with PBS solution. It was stored in 0.5% GA solution at 4°C until use.

### 2.5 Main reagents and instruments

#### 2.5.1 Mesenchymal cell (MSC) cultures

**Figure 1:**
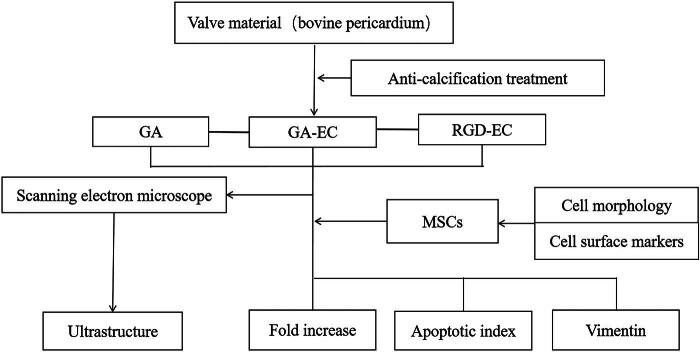
Protocol of the experiment.

The bilateral back femur bone was removed from 4-week-old male SD rats under sterile conditions. Dulbecco’s modified Eagle’s medium-low glucose (DMEM-LG) was used to flush the marrow cavity. The cell suspension was filtered through a 200-mesh sieve to collect the single cells. They were then washed two times with PBS. The cells were then suspended in fetal calf serum (FCS) DMEM-LG (15%) and stored in a plastic bottle (50 ml). Cell crossbreeding and amplification were performed in a cell culture box at 37°C, 5% CO_2_, and 100% relative humidity. The cells were cultured for 48 h, and the medium was renewed every 3–4 days as follows. When the cells covered approximately 80% of the bottom of the culture flask, they were digested using trypsin with 0.25% ethylenediaminetetraacetic acid and cultured at a ratio of 1:3. When MSCs were cultured to the third generation, they were identified, and related experiments were performed (Figure 1).

### 2.6 Identification of mesenchymal stem cells using flow cytometry

MSCs express various surface markers on stromal cells, endothelial cells, epidermal cells, and other cells. It is generally believed that CD29, CD44, CD90, and CD105 are important surface markers of MSCs. As MSCs are non-hematopoietic cells, the expression of hematopoietic cell surface antigens, such as CD34 and CD45, is negative. The third generation of MSCs was prepared as a total of 2 × 10^6^ single-cell suspensions. A total of 100 μl suspension was incubated with CD44, CD45, and CD90 monoclonal antibodies. After washing three times with PBS, the cells were incubated with FITC-labeled IgG antibody for 30 min. After washing three times with PBS, the cells were identified using flow cytometry. The same negative control was used for each sample. The flow cytometry results were analyzed using CellQuest software (Becton Dickinson and Company, Franklin Lakes, NJ, USA), especially the purity and properties of the *in vitro* cultured MSCs.

### 2.7 Morphology of mesenchymal stem cells grown on different biological valves

The morphology of MSCs grown in biological valves, each of which was exposed to one of the three treatment techniques, was determined using a scanning electron microscope. The valves were moved from GA to PBS for 3 days, during which the PBS solution was changed daily. The valves that had been placed in PBS were irradiated with ^60^Co. After irradiation, sterile PBS was changed every day for 3 days. Each valve was then placed in a 6-well plate, and MSCs were grown until the number of cells in each well was 5 × 10^4^. The PBS medium was changed every 3 days. The culture was maintained for 1 week, and the medium was aspirated. The cells were washed twice with PBS and fixed with 3% GA for 12 h. SEM specimens were further prepared and observed using a scanning electron microscope (HitachiS-3400N, Hitachi Limited, Japan).

### 2.8 Detection of cell adhesion ability

Third-generation MSCs were selected from the logarithmic growth phase. About 1 × 10^5^ single cells from the third-generation MSC suspension were injected into different valve materials placed in 6-well plates. After culturing for 1 week, all valve materials on which the MSCs were grown were placed in a Rectangular Flow Chamber (Glycotech Corporation, USA) and treated with 10 dyn/cm^2^ shear stress for 24 h. Cells (1 × 10^5^/ml) from each group were cultured in 6-well plates for 8 h. The cells were then dispersed in 0.25% trypsin and counted. The adherent rate was calculated using the following formula: adherent rate = (number of adherent cells/number of inoculated cells) × 100%. The values are represented in %.

### 2.9 Preparation of cells using the terminal deoxynucleotidyl transferase deoxyuridine triphosphate nick end labeling method and the expression of vimentin in mesenchymal stem cells by immunofluorescence

Valve materials with adhered MSCs that had received 10 dyn/cm^2^ shear stress intervention for 24 h and dispersed were grown until the cells covered approximately 80% of the surface of the valve material. The cells were fixed with 4% paraformaldehyde for 30 min. The cells were then divided into two groups. One group was subjected to the TUNEL assay, and the other group was tested for vimentin expression in MSCs by immunofluorescence.

### 2.10 Cells subjected to the terminal deoxynucleotidyl transferase deoxyuridine triphosphate nick end labeling method

The cells were washed in PBS solution and incubated with 0.1% Triton X-100 for 5 min. After washing with PBS solution, TUNEL liquid was added for detection. The cells were incubated in the dark at 37°C for 60 min before treating with DAPI and incubated in the dark at 37°C for 5 min. The cells were mounted with 50% glycerol and observed under an Olympus Fluoview microscope (FV1000, Olympus, Japan). The images were obtained, and statistical analysis was performed.

### 2.11 Expression of vimentin in mesenchymal stem cells by immunofluorescence

Cells from each group were washed with PBS and incubated with 3% H_2_O_2_ at room temperature for 10 min. After washing in PBS (5 min, 3 times), they were treated with 5% normal goat serum and incubated at room temperature for 10 min. Then, the serum was removed. A 1:100 dilution of rabbit anti-mouse vimentin was incubated overnight at 4°C. After washing in PBS, the FITC-labeled goat anti-rabbit IgG antibodies were diluted to 1:1,000. The cells were incubated in the dark at room temperature for 2 h and washed with PBS. The cells were stained with DAPI for 5 min and washed with PBS. They were then mounted with 50% glycerol and observed under an Olympus Fluoview microscope (FV1000, Olympus, Japan). Images were obtained for counting, and statistical analysis was performed. At the same time, for the control, the primary antibody was replaced with PBS solution and incubated.

### 2.12 Statistical analyses

Statistical analyses were conducted using the SPSS software (version 22.0; IBM Corp, Armonk, NY, USA). Continuous variables were presented as mean ± SD, and categorical variables were expressed as percentages. Univariable comparisons were performed using the Student’s unpaired *t*-test for continuous normally distributed data and the *χ* ^2^ test for categorical data. Statistical significance was set at *P* < 0.05.


**Ethical approval:** The experimental procedures involving animals were performed in accordance with the guidelines of the Ethics Committee of Xijing Hospital, The Fourth Military Medical University (No. KY20212224-C-1) and local laws and policies. All protocols were responsible for our research organization(s) following all guidelines, regulations, and legal and ethical standards as required for humans and animals.

## 3 Results

### 3.1 Morphology of the cultured primary mesenchymal stem cells

**Figure 2:**
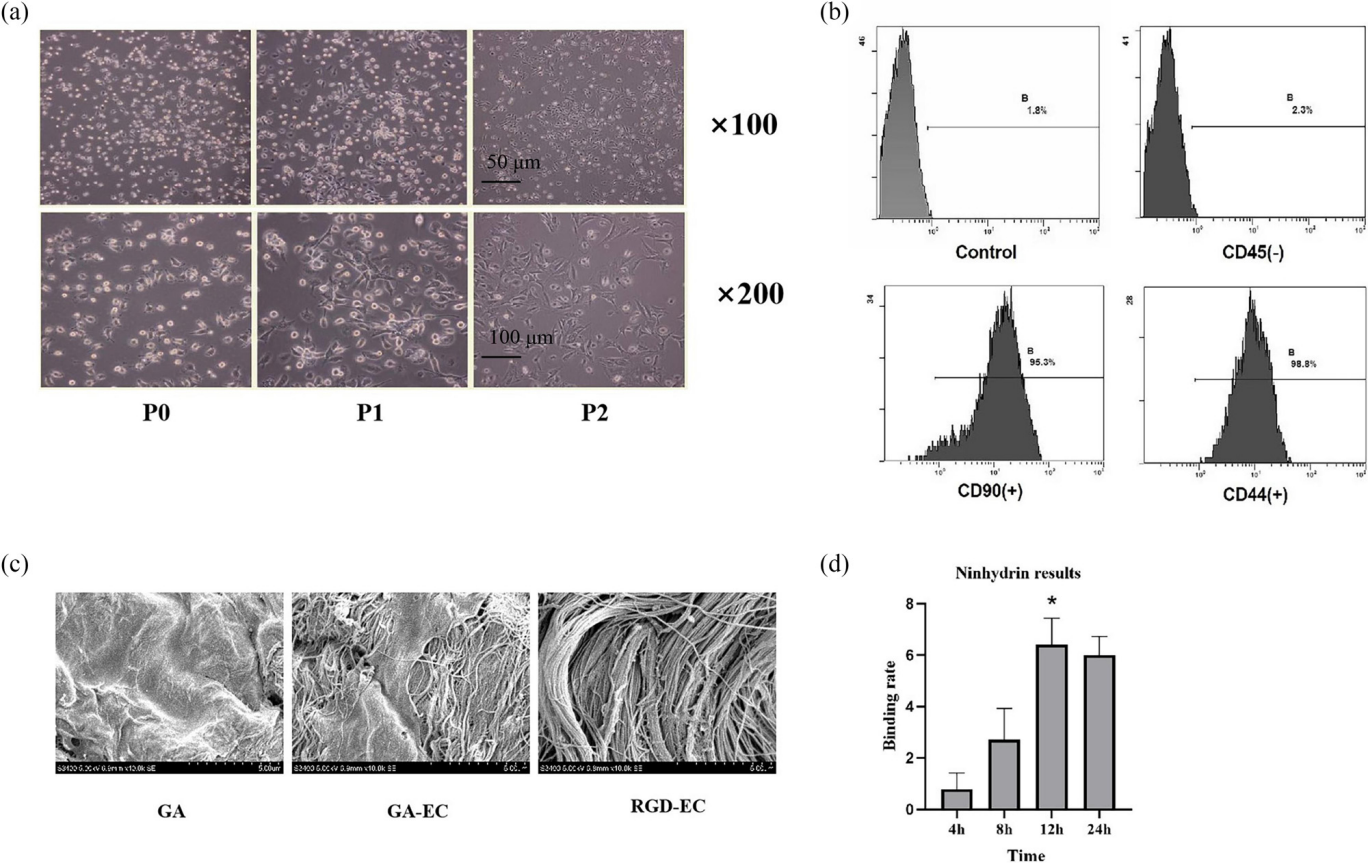
Identification of bone marrow mesenchymal stem cells and detection of RGD-EC treatment bovine pericardium. (a) Cell morphology of the primary cultured MSCs. (b) Identification of MSCs using flow cytometry. (c) Ultrastructure of the the smooth surface on the endocardial side of the bovine pericardium after different treatments (scanning electron microscope, ×10,000). (d) Results of the ninhydrin experiment (*n* = 3, *P < 0.05 vs 4 h group).

Primary cultured MSCs were observed under a microscope (Figure 2). The cells suspended in the culture medium were round, and the nuclei were located in the cell centers. After incubating for 1–2 h, the cells began to adhere to the wall. After 24 h, most cells were attached to the walls. Approximately 10 days later, each colony contained 100–200 cells. Cell morphology was homogeneous; some cells assumed a fibroblast-like spindle shape, occasionally with large, flattened polygonal cells. After about 12–14 days, the cells fused into a single layer. Passaging cells were attached to the wall within about 0.5–1 h and were completely adherent within 24 h. The cells had wide and flat polygons. After 5–6 days, the cells fused completely into uniform fusiform shapes. The characteristics of primary cultured cells were consistent with those of MSCs.

### 3.2 Primary cultivated mesenchymal stem cells using flow cytometry

The expression rate of CD45 on primary cultured MSCs was 2.3%, that of CD90 was 95.3%, and that of CD44 was 98.8%. These results are consistent with the expression of markers on the MSC cell surface [18].

### 3.3 Scanning electron microscopy

Scanning electron microscopy results showed that the surface morphology of the valve materials varies with different surface anti-calcification treatments. The surface of the valve in the GA treatment group was relatively flat, with a microporous structure, and the surface of the valves in the GA-EC and RGD-EC treatment groups had a filamentous structure with a small number of protein components attached to the culture medium.

### 3.4 Terminal deoxynucleotidyl transferase deoxyuridine triphosphate nick end labeling for apoptosis detection

**Figure 3:**
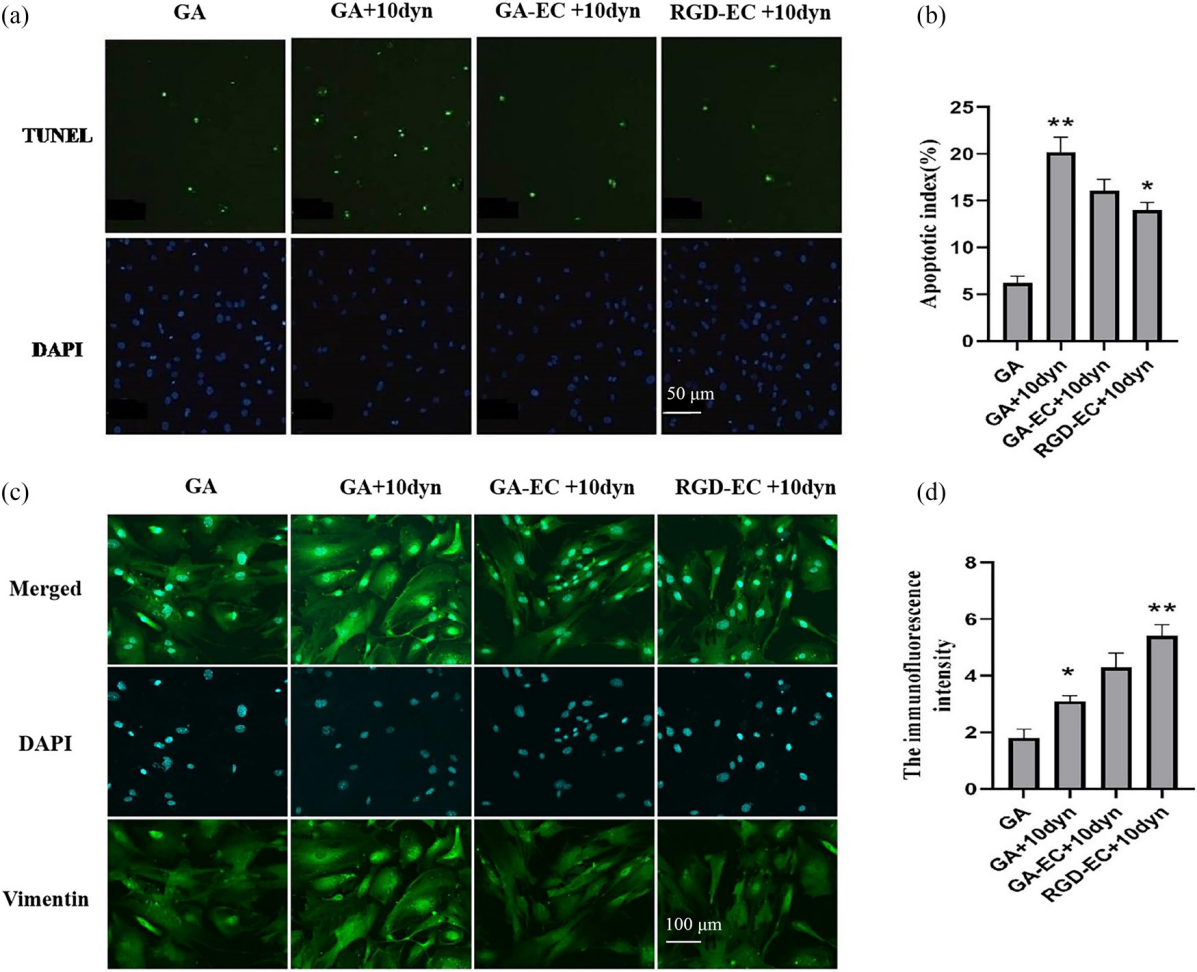
Differences of valve materials in each group after exposed to a shear stress of 10 dyn/cm^2^. (a) TUNEL staining images for each group (×200). (b) The apoptotic index of each group (*n* = 3, *P < 0.05 vs GA group, **P < 0.01 vs GA + 10 dyn group). (c) Differences in the extent of vimentin expression in the different valve groups after exposed to a shear stress of 10 dyn/cm^2^ (×400). (d) The immunofluorescence intensity of each group (*n* = 3, *P < 0.05 vs GA group, **P < 0.01 vs GA + 10 dyn group).

After being subjected to shear stress (10 dyn/cm^2^), the apoptosis rate of MSCs in the GA group was significantly higher than that in the GA group (*P* < 0.01). After being subjected to shear stress (10 dyn/cm^2^), the apoptosis rate of cells in the GA + EC and RGD + EC groups was lower than that in the GA group. The apoptosis rate of RGD + EC cells was significantly lower than that of the GA + 10 dyn group (*P* < 0.05), whereas no significant difference was evident compared to the GA group (Figure 3).

### 3.5 Adherent rate detection

Compared to the GA group, which was subjected to shear stress (10 dyn/cm^2^) after the intervention, there was no significant difference in the GA + EC group. The adhesive ability of MSCs in the RGD + EC group was significantly higher than that in the GA group (*P* < 0.05).

### 3.6 Expression of vimentin

The results of immunofluorescence staining showed that the conventional static culture of MSCs did not express the smooth muscle cell marker vimentin. The results of vimentin staining were negative in most MSCs. After the cells were subjected to a shear stress of 10 dyn/cm^2^, the test results for vimentin were positive in the different groups. The expression of vimentin was increased in the GA + EC, GA, and GA groups. The expression of vimentin increased significantly in the RGD + EC group compared to that in the static control group (*P* < 0.05).

## 4 Discussion

Approximately 300,000 cases of aortic valve calcification occur annually in patients who may need aortic valve replacement worldwide. In China, the proportion of elderly patients with degenerative and calcific valvular disease has increased [18]. This is mainly a reason for cardiac valve replacement in elderly patients [19]. With the development of biological techniques, valve replacement has become an additional choice for valvular heart diseases. However, problems related to poor long-term durability, easy calcification, and other shortcomings exist in biological valves.

Traditional methods for modifying the surfaces of artificial heart valves include surfactants, competitive inhibitors, chemical modifiers, cross-linking modifications, and the construction of tissue-engineered heart valves. It requires that the biological valve material not only meets the requirements of the traditional valve but also has the required mechanical strength, valve morphological stability, tear strength, a conveying sheath with a small diameter, anti-calcification properties, and other special requirements. Our study combines the characteristics of independent research and the development of new heart valves that combine the oxidation of the low-molecular-weight heparin/antithrombin complex with GA to modify the surface of the biological valves. Furthermore, it uses an RGD peptide on the GA surface and a bionic pulsating bioreactor to construct a tissue-engineered biological valve. It was designed to improve the properties of biological valve mechanics in terms of anti-calcification activity, biocompatibility, and long-term durability.

Extracellular biomechanical signals are important for the regulation of growth and development of processed heart valves. They allow the valve to adapt gradually to the hemodynamic environment under high pressure and high speed of cell development. It is critical to systematically simulate mechanical signal regulation processes, such as gradually increasing pressure and blood flow velocity, during valve physiological development.

MSCs have the advantages of multidirectional differentiation, low immunogenicity, convenience, and ease of amplification *in vitro*. In 2002, MSCs were introduced in the field of cardiovascular tissue engineering and were used as seed cells in scaffold materials in 2005. The main standard for the identification of primary cultured MSCs is that the cell surface molecule CD45 is negative, and CD44 and CD90 are positive [20,21]. As a member of the transmembrane glycoprotein family, CD45, also called leukocyte common antigen (LCA), exhibits protein tyrosine phosphatase activity and is expressed in leukocytes. CD90, also known as Thy-1, is expressed in mature cells as a glycosyl phosphatidyl inositol surface glycoprotein, mainly in the brain and lymphoid tissues. MSCs showed a high expression of CD90. CD44 is associated with adhesion molecules and is highly expressed in mesenchymal MSCs. The characteristics of the primary MSCs cultured in this study were consistent with those mentioned above.

Our preliminary study showed that 0–15 dyn/cm^2^ progressive mechanics not only improved the active function of MSCs in older rats and reduced apoptosis but also induced MSCs *in vitro* to differentiate in the direction of smooth muscles. Therefore, in this study, shear stress parameters of 10 dyn/cm^2^ were used.

According to prior experience accumulated in our laboratory, it not only enhanced cell adhesion, proliferation, and differentiation ability but also exhibited structural and mechanical properties of better organization to modify the decellularization scaffold with the treatment of the RGD peptide and EC. Therefore, we emphasize the importance of further exploring the application of the RGD polypeptide combined with EC surface modification technology and a bionic pulsating bioreactor to construct a metabolism with the ability to repair itself and tissue-engineered valve materials and to provide a new idea to fundamentally solve the tendency of the biological valve to fail, the problem of calcification, and the need to develop new biological valve materials. The results of the current study showed that GA-EC, RGD-EC, and anti-calcification valvular surfaces have a filamentous structure and that more cell metabolites adhere to the surface. The morphology of the MSCs showed that the GA-EC and RGD-EC groups and the anti-calcific valvular surfaces were more compatible with adhesive and biological valve materials, and our laboratory results were consistent with those of others [22,23]. On dynamic flow conditions, the different anti-calcification groups were affected by the shear stress (10 dyn/cm^2^). Comparing the GA-EC, RGD-EC, and GA groups with the traditional adhesive ability of the cells, the cell apoptosis rate decreased, as did the expression of vimentin.

Above all, treatment with RGD and EC could significantly improve the biological characteristics of the valve surface and provide a theoretical basis for further study of biological cardiac valves. This may be helpful in solving the issue of poor long-term durability associated with biological valve materials and provide new ideas for the research and development of new biological valve materials. By surface modification with the RGD peptide, sufficient contact between the scaffold and seed cells was ensured, which promoted cell and extracellular matrix proliferation. Due to limitations in conditions, the control of the reaction conditions for short peptides and bovine pericardium in this experiment was not precise enough, and the molecular mechanisms of cell and extracellular matrix growth also need to be further explored.
